# Stratified management of residual gastric cancer risk after *Helicobacter pylori* eradication

**DOI:** 10.3389/fmicb.2026.1779490

**Published:** 2026-02-13

**Authors:** Li Song, Qi-Ying Yu

**Affiliations:** 1 Central Laboratory and Department of Oncology, Tumor Hospital Affiliated to Nantong University, Nantong, Jiangsu, China; 2 School of Biological Science and Engineering, Southeast University, Nanjing, Jiangsu, China

**Keywords:** artificial intelligence, biomarkers, gastric cancer, gastric microbiome, *Helicobacter pylori* eradication, OLGA/OLGIM, oncogenic memory, precision prevention

## Abstract

Despite the established efficacy of *Helicobacter pylori* eradication in reducing gastric cancer (GC) incidence, a significant residual risk persists in successfully treated individuals, driven by lasting pathological alterations termed “oncogenic memory,” including irreversible mucosal damage (e.g., intestinal metaplasia), residual pro-inflammatory and epigenetic “molecular scars,” and gastric microbiome dysbiosis. This perspective synthesizes current evidence to advocate for a paradigm shift from a singular focus on pathogen clearance towards a comprehensive, risk-adapted management strategy. We propose a novel, dual-dimensional framework centered on a multidimensional risk assessment that integrates OLGA/OLGIM staging, demographic, lifestyle, and genetic factors to stratify post-eradication individuals into distinct risk categories. The framework subsequently outlines tailored surveillance protocols—specifying endoscopy frequency and advanced biomarker application—leverages technological support from AI-assisted endoscopy and molecular testing, and details differentiated resource allocation models based on regional GC incidence and economic development. This integrated approach provides a practical roadmap for implementing precision prevention, aiming to mitigate the lingering GC risk and ultimately reduce the global disease burden through a dynamic, lifelong management system beyond eradication. To facilitate implementation, we provide a user-ready risk calculator that operationalizes the multidimensional score for cohort-level application.

## Graphical abstract

**Figure:**
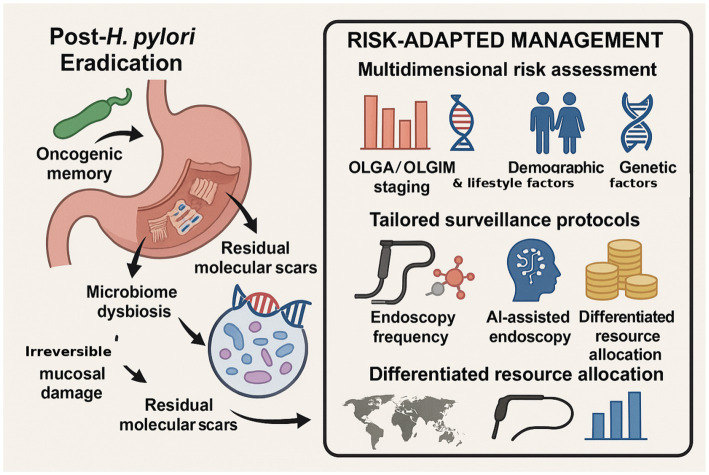
Graphical summary of the dual-dimensional framework for post-Helicobacter pylori precision prevention. Despite successful H. pylori eradication, residual gastric cancer risk persists due to long-lasting pathological and molecular alterations collectively referred to as oncogenic memory. These include irreversible mucosal damage (e.g., intestinal metaplasia), persistent epigenetic and inflammatory molecular scars, and gastric microbiome dysbiosis. The proposed dual-dimensional management framework integrates a multidimensional risk assessment—encompassing OLGA/OLGIM staging, demographic, lifestyle, and genetic factors—with tailored surveillance protocols, including optimized endoscopy frequency, AI-assisted endoscopic imaging, and application of advanced molecular biomarkers. Furthermore, differentiated resource allocation models are recommended according to regional gastric cancer incidence and economic capacity. This precision prevention approach aims to transform post-eradication management from simple pathogen clearance to a dynamic, lifelong, risk-adapted strategy.

## 1 Introduction

*Helicobacter pylori* (*H. pylori*) infection is the most significant modifiable risk factor for gastric cancer, and its eradication has been demonstrated to reduce gastric cancer incidence by 63%, establishing it as a cornerstone of global gastric cancer prevention strategies (Li et al., 2023). However, emerging evidence from clinical studies challenges the conventional treatment endpoint by revealing that a subset of individuals successfully treated for *H. pylori* continue to exhibit a substantial residual risk of gastric cancer (Wiklund et al., 2025; Yamada et al., 2025). This phenomenon underscores the limitations of a pathogenetic prevention model for addressing the complex pathogenesis of gastric cancer.

The underlying biological mechanisms are becoming increasingly well defined. Long-term *H. pylori* infection leaves behind a form of “oncogenic memory” in the gastric mucosa, which is characterized by persistent alterations in the microenvironment, accumulated epigenetic modifications, and sustained low-grade chronic inflammation. These changes may continue to drive carcinogenesis, even after bacterial clearance. Furthermore, multiple risk pathways independent of *H. pylori*, such as high-salt diets, smoking, alcohol consumption, genetic susceptibility, and preneoplastic conditions, including gastric atrophy and intestinal metaplasia, collectively contribute to a multidimensional risk profile for gastric cancer development.

In response, we advocate a shift in gastric cancer control from a singular focus on bacterial eradication to comprehensive, multidimensional risk management. Artificial intelligence (AI) can enable this transition. In resource-limited settings, AI-assisted portable endoscopy, mobile risk-assessment tools, and remote image interpretation can support standardized screening and referral. In high-risk regions with stronger resources, AI can be combined with multi-omics biomarkers and advanced image analytics to build dynamic risk-prediction models and support precision prevention. Together, these components can form an integrated prevention network that moves beyond pathogen clearance to lifelong, risk-adapted management.

## 2 Biological mechanisms reveal the root causes of residual risks

### 2.1 Persistent mucosal injury

Atrophic gastritis and intestinal metaplasia often persist after *H. pylori* eradication. Although eradication halts the infection-driven “Correa cascade” (from inflammation to atrophy, metaplasia, and ultimately dysplasia), established preneoplastic lesions are typically irreversible, with intestinal metaplasia showing minimal regression (Borka Balas et al., 2022). Meta-analyses indicate that eradication at this stage does not significantly reduce gastric cancer risk, representing a critical “point of no return” (Machlowska et al., 2020). The occurrence of early-onset cancer after eradication in some patients underscores that mucosal progression can continue despite bacterial clearance.

### 2.2 Residual immunological and epigenetic alterations

Long-term *H. pylori* infection induces immunological and epigenetic changes that resolve slowly. *H. pylori*-specific Th17 cells and elevated pro-inflammatory cytokines such as interleukin-1β may persist, sustaining a state of low-grade inflammation (Serelli-Lee et al., 2012). Furthermore, bacteria-induced epigenetic alterations—including hypermethylation of tumor suppressor genes—can endure in epithelial cells as “molecular scars.” These alterations continue to promote tumorigenesis until the affected cells are replaced through normal turnover (Thrift et al., 2023).

### 2.3 Gastric microbiome dysbiosis

Eradication therapy alters the gastric microbiota, which may subsequently promote the colonization of bacteria capable of producing carcinogens such as N-nitroso compounds. This risk is particularly pronounced in individuals with atrophic gastritis (Li et al., 2017). Patients with severe baseline mucosal damage often experience prolonged dysbiosis, maintaining pro-inflammatory and carcinogenic microbial profiles, whereas those with healthier mucosa exhibit more rapid recovery (Salvatori et al., 2023). This disparity suggests that eradication alone may be insufficient to restore a healthy gastric microenvironment in high-risk individuals.

### 2.4 Synthesis and implications

Collectively, these mechanisms demonstrate that the risk of gastric cancer persisting after *H. pylori* eradication originates primarily from irreversible pathological changes. This evidence highlights the necessity to address the often-overlooked issue of “post-eradication risk” and to reorient prevention strategies from a binary focus on “whether eradication was achieved” to a more nuanced assessment of “what risks remain,’’ including *H. pylori* recurrence (Hu et al., 2017) To facilitate this paradigm shift, we have systematically reviewed the key risk factors, synthesizing them in Table 1 to inform risk stratification and enable precision monitoring in post-eradication populations. Based on this framework, clinical pathways should integrate these multidimensional risks to guide actionable interventions. Accordingly, we propose the following stratified management strategy.

**Table 1:** Risk-based management strategies for gastric cancer following *Helicobacter pylori* eradication.

Risk dimension	Core factor	Risk impact and mechanism	Precision prevention strategy	Primary care implementation pathway
Demographics	Age	Younger population (≤55 years): Higher standardized incidence ratio (SIR) post-eradication, potentially associated with detection bias and baseline risk (Li et al., 2023; Wiklund et al., 2025; Thrift et al., 2023)	Establish a refined, age-stratified management system	Develop age-stratified health record repositories
		Older population (>70 years): Significant relative benefit, but requires comprehensive assessment of life expectancy and competing risks from comorbidities (Li et al., 2023; Wiklund et al., 2025; Thrift et al., 2023)	Conduct individualized benefit–risk assessment	Conduct targeted health education on gastric cancer risk
				Implement comprehensive geriatric assessment and life expectancy evaluation.
	Gender	Male risk 2–3 times higher than female: Attributable to sex hormone differences, lifestyle factors, and healthcare-seeking behavior (Machlowska et al., 2020; Thrift et al., 2023)		Prioritize gastric cancer screening programs for males.
			Implement sex-specific follow-up	Conduct male-focused health promotion campaigns
				Implement sex-specific follow-up
Pathological changes	Gastric mucosal status	Atrophic gastritis/intestinal metaplasia: Persistent precancerous lesions; intestinal metaplasia represents an irreversible point (Mera et al., 2018; den Hoed et al., 2013; Miki, 2011)	Perform standardized OLGA/OLGIM staging assessment prior to eradication (den Hoed et al., 2013; Miki, 2011)	Promote initial screening with gastric function serology tests (PG I/II, G-17) (Pimentel-Nunes et al., 2019; Dinis-Ribeiro et al., 2012)
		Correa cascade: The pathological mucosal environment may still progress towards malignancy post-eradication (Machlowska et al., 2020; Salvatori et al., 2023; Mera et al., 2018)	Develop individualized management plans based on pathological staging	Establish pathways for identifying and referring high-risk individuals
				Deliver health management guidance for precancerous conditions
Temporal factors	Time since eradication	Risk persistence up to 11 + years: Due to slow resolution of epigenetic alterations and immunological abnormalities (Li et al., 2023; Wiklund et al., 2025; Yamada et al., 2025; Serelli-Lee et al., 2012)	Establish a lifelong, continuous risk monitoring system	Improve the electronic health record system for residents
		Delayed protective effect: Time required for molecular “scar” repair (Yamada et al., 2025; Salvatori et al., 2023)	Develop dynamically adjusted follow-up schedules	Implement standardized long-term follow-up management
				Create automated reminder and appointment systems
Medication influences	PPI use	Independent risk factor (HR = 1.5–2.0): Long-term use alters gastric environment, potentially promoting tumorigenesis (Cheung et al., 2018)	Enhance endoscopic surveillance for long-term users	Conduct education on rational medication use
				Establish specialized management for long-term PPI users
				Monitor for adverse drug reactions and intervene
	Statin drugs	Potential chemopreventive effect: Protective via anti-inflammatory, cell cycle regulation, and other pathways (Gutierrez-Chakraborty et al., 2024)	Systematically document medication history and assess protective efficacy	Integrate into comprehensive chronic disease management
			Explore combination chemoprevention strategies	Evaluate synergy between cardiovascular and cancer prevention benefits
Lifestyle	Smoking, alcohol, diet	Established risk factors: Tobacco, alcohol, high-salt and preserved foods directly damage gastric mucosa and synergistically promote carcinogenesis	Systematically collect lifestyle information	Promote healthy lifestyles in the community
		Cumulative effect: Long-term adverse habits significantly increase risk (Machlowska et al., 2020; Thrift et al., 2023)	Formulate personalized behavioral intervention plans	Establish a special intervention plan for quitting smoking and limiting alcohol consumption
				Promote balanced nutrition and low-salt diets (Machlowska et al., 2020; Thrift et al., 2023)
	Family history	Significantly increased risk with first-degree relatives: Combined effect of genetic susceptibility and shared environmental factors (Wang et al., 2023)	Must be included as a core risk assessment indicator	Improve registry and reporting of family tumor history
			Establish early warning for familial clustering cases	Flag high-risk families for focused management
				Offer genetic counseling services
Microbial factors	Strain characteristics	cagA-positive strains: Higher virulence, significantly increased carcinogenic risk (Ligato et al., 2024; Salvatori et al., 2023)	Promote virulence typing of strains	Implement *H. pylori* screening programs
		Reinfection risk: Particularly in high-prevalence areas, requiring ongoing attention (Huang et al., 2003)	Implement post-eradication confirmation and periodic (den Hoed et al., 2013; Miki, 2011; Huang et al., 2003)	Manage post-eradication confirmation testing (den Hoed et al., 2013; Miki, 2011; Huang et al., 2003)
				Conduct education on reinfection prevention
	Gastric microbiome	Dysbiosis promoting carcinogenic environment: Post-eradication microbial imbalance, proliferation of carcinogen-producing bacteria (Li et al., 2017; Salvatori et al., 2023)	Explore gastric microbiome analysis as a biomarker (Li et al., 2017; Salvatori et al., 2023)	Promote the application of probiotics and other microecological preparations (Su et al., 2022; Yang et al., 2024)
		Long-term impact: Slow recovery in individuals with severe mucosal damage	Investigate microbiome modulation strategies	Carry out health education on dietary fiber and prebiotics
				Monitor microbiome recovery
Resource allocation	Regional differences	Significant epidemiological variation: Different risk factor distributions in high vs. low incidence regions (Thrift et al., 2023)	Develop region-specific prevention systems	Design prevention programs based on regional characteristics
		Resource inequality: Regional disparities in healthcare access and technical capacity (Thrift et al., 2023)	Optimize resource allocation and utilization efficiency	Establish tiered diagnosis/treatment and two-way referral systems
				Conduct specialized training for primary care staff

## 3 A dual-dimensional framework for precision prevention: a practical approach

Confronted with the challenge of “residual risk after eradication, the conventional “one-size-fits-all” prevention model is insufficient. It is imperative to establish a dual-dimensional precision prevention system that integrates risk assessment with stratified interventions to achieve “comprehensive risk coverage and individualized intervention” as the modern goal of gastric cancer prevention and control.

### 3.1 Risk assessment: building a dual-core model integrating *Helicobacter pylori* status and multidimensional factors

This framework moves beyond traditional assessments based solely on *H. pylori* infection status. It innovatively integrates *H. pylori* status (positive, negative, or eradicated) with multidimensional risk factors, including age, sex, severity of gastric mucosal lesions, family history, lifestyle, medication history, and comorbidities to establish a dual-core assessment model.

After *H. pylori* eradication, core assessment indicators include OLGA/OLGIM staging (evaluating the degree of mucosal atrophy and intestinal metaplasia), sex (higher risk in males), age (≥50 years defined as high-risk), and history of proton pump inhibitor (PPI) use (≥3 years considered high-risk) (Cheung et al., 2018). Based on the assessment results, this population was stratified into three categories: *very high-risk* (OLGA/OLGIM stages III–IV plus at least one other high-risk factor), *high-risk* (OLGA/OLGIM stages III–IV alone OR OLGA/OLGIM stages I–II plus at least one other high-risk factor), and *low-to-moderate-risk* (OLGA/OLGIM stages 0–II without additional high-risk factors) (Mera et al., 2018; den Hoed et al., 2013).

### 3.2 Monitoring and intervention: implementing a dual-path strategy with stratification and differentiation

To facilitate immediate clinical translation, we provide (i) a mock-up risk calculator in Excel (Supplementary File S1) and (ii) a lightweight web version (Supplementary File S2, HTML) that operationalize the proposed multidimensional scoring framework and allow readers to input their own cohort characteristics to obtain an illustrative risk tier. Based on the results of the dual-core assessment, we formulated differentiated monitoring and intervention plans to ensure efficient allocation of medical resources.

Very high-risk individuals post-eradication are recommended to undergo annual meticulous endoscopy (including magnifying endoscopy combined with narrow-band imaging and chromoendoscopy) coupled with testing for serum RIMS1 methylation levels to assess molecular residual risk (Yamada et al., 2025). Probiotic interventions to modulate microbial imbalances were considered necessary.

High-risk individuals post-eradication are recommended to undergo biennial “serological testing (Gastrin-17, Pepsinogen I/II ratio) plus meticulous endoscopy.” If serological markers are abnormal, monitoring frequency should be increased annually (den Hoed et al., 2013; Pimentel-Nunes et al., 2019; Miki, 2011; Dinis-Ribeiro et al., 2012; Robles et al., 2022).

High-risk *H. pylori*-negative individuals are recommended to undergo meticulous endoscopy every 2–3 years, combined with lifestyle questionnaire assessments, reinforced with smoking cessation and low-salt diet guidance.

Low-to-moderate-risk individuals (including both post-eradication and *H. pylori*-negative individuals) are recommended to undergo serological screening every 3–5 years. Endoscopy should be initiated if the results are abnormal, along with the promotion of healthy lifestyles through community-based health education.

Alignment with existing guidelines: Our framework is designed to complement—rather than duplicate—current guideline-based prevention strategies. The Kyoto Global Consensus highlights the importance of grading systems for gastric cancer risk stratification and endorses image-enhanced endoscopy for gastritis assessment (Fontes et al., 2025). MAPS-II provides evidence-based surveillance intervals for patients with atrophic gastritis and intestinal metaplasia, including OLGA/OLGIM-based risk categories (den Hoed et al., 2013). Building on these foundations, we extend guideline stratification by integrating post-eradication “oncogenic memory,” host genetics, lifestyle/exposure factors, and emerging AI/biomarker tools to support more individualized, resource-aware precision prevention.

### 3.3 Technology empowerment: building a dual support system of “artificial intelligence + biomarkers”

Technological innovation serves as the core driver for enhancing the precision and accessibility of gastric cancer prevention and control. By integrating artificial intelligence with cutting edge biomarker detection, we can construct a precise prevention and control system that covers the entire process. This system not only significantly improves the identification efficiency of early lesions, but also effectively bridges the gap in diagnosis and treatment levels across different regions, forming a technological cornerstone for revolutionizing gastric cancer control (Sugano et al., 2015; Hirasawa et al., 2018; Luo et al., 2019; Wu et al., 2021; Miki, 2006).

#### 3.3.1 Innovation and application of artificial intelligence technology

##### 3.3.1.1 Advanced breakthroughs in endoscopic image analysis

AI in endoscopic image analysis has transcended mere lesion identification and entered a new stage of precise segmentation and quantitative analysis. For instance, a recent study by a team from Fudan University developed an AI assisted ratio responsive Raman array system for visualizing mucosal acidity changes during endoscopy. This technology utilizes a multimodal neural network to analyze Raman spectra and accurately distinguish early gastric cancer from inflammatory tissues. In an external validation involving 389 sampling points, a comprehensive accuracy of 86.89%, sensitivity of 87.79%, and specificity of 85.04% were achieved (Wang et al., 2020). This signifies that AI technology can now integrate morphological and biochemical information, evolving from “seeing” to “insight,” providing unprecedented technical support for precisely defining the scope of endoscopic submucosal dissection (ESD).

##### 3.3.1.2 A revolutionary noninvasive screening paradigm: “AI + plain CT”

The DAMO GRAPE model, jointly developed by Zhejiang Cancer Hospital and Alibaba DAMO Academy, was the world’s first AI model for gastric cancer imaging screening. Its research findings have been published in the prestigious international journal, Nature Medicine. The groundbreaking significance of this model lies in its successful implementation of early gastric cancer screening using widely available, low-cost, and noninvasive plain CT, turning the “impossible” into “possible.”

*Exceptional performance*: The model demonstrated a sensitivity of 85.1% and specificity of 96.8%, significantly surpassing the diagnostic level of radiologists.

*Paradigm value*: It innovatively proposes a new pathway: “plain CT initial screening + AI risk stratification + targeted gastroscopic examination.” This approach reduces the proportion of the high-risk population requiring gastroscopy from 20%–25% identified by traditional questionnaire screening, to approximately 6%. In simulated trials, the gastric cancer detection rates reached 24.5 and 17.7%, respectively, far exceeding the traditional rate of 1.16%. This significantly optimizes the allocation efficiency of medical resources.

*Prospective early warning*: Retrospective analysis indicated that this AI model could detect subtle signs of gastric cancer in plain CT images 2–10 months in advance, securing a crucial window for “early diagnosis and treatment” (Yan et al., 2025).

#### 3.3.2 The central role of AI in narrowing regional disparities in diagnosis and treatment

The inherent characteristics of AI technology—replicability, standardization, and boundary less access via cloud computing—make it a key tool in addressing the uneven distribution of medical resources (Hu et al., 2025; Monteiro et al., 2016).

##### 3.3.2.1 Empowering primary care: from technology deployment to capacity building

*Standardized diagnostic output*: AI models can package the diagnostic expertise of high-volume centers into algorithms that are deployable in primary care. Deep learning systems can analyze endoscopic images in real time and identify precancerous lesions such as atrophy and intestinal metaplasia with high accuracy. Transfer learning also enables rapid local adaptation using small amounts of site-specific data, helping to standardize diagnostic workflows across regions (Verbraak et al., 2019; Shi et al., 2023).

*Remote intelligent collaboration*: Cloud platform-based AI diagnostic systems enable primary hospitals to upload imaging data and receive AI-assisted analysis reports from superior centers within a short time, constructing a “digital medical highway” for the instant sharing of premium diagnostic resources.

##### 3.3.2.2 Reshaping screening pathways for precision and equity

The success of the DAMO GRAPE model represents not only a technological breakthrough but also an innovation in public health screening models. It utilizes the already widespread availability of plain CT equipment for “opportunistic screening,” allowing individuals to simultaneously undergo low-cost initial gastric cancer risk assessment during CT scans performed for other reasons (e.g., pulmonary nodules), without the additional discomfort of gastroscopy or high costs (Yan et al., 2025). This “one plain CT scan, multiple cancer screenings” model significantly lowers the barrier for largescale population screening, offering a “Chinese solution” tailored to national conditions for implementing efficient gastric cancer control in regions with relatively limited medical resources.

#### 3.3.3 Deep integration of biomarkers and AI

##### 3.3.3.1 Refinement of biomarkers and AI driven rediscovery

In the field of biomarkers, AI plays a dual role as both a “miner” and “integrator.”

*Discovery of novel markers*: Beyond traditional serological markers, research has shifted towards more microscopic levels. For instance, a study by a Japanese team published in Gut confirmed the level of RIMS1 gene methylation in gastric mucosa with persistent atrophy post *H. pylori* eradication is a powerful risk predictor, with the high-risk group having a cancer risk 470% higher than that of the low-risk group (Yamada et al., 2025).

*AI mining “legacy data”*: The power of AI lies in its ability to uncover new values from previously overlooked data. A liver study published in the JHEP Reports provides an excellent paradigm: researchers used modern AI classification models such as Random Forest and Decision Tree to reanalyze a 2017 extracellular vesicle (EV) dataset. They successfully identified a rare EV subpopulation (AnnV+EpCAM+CD133+gp38+), which improved the accuracy of liver cancer detection from the original AUC of 0.70 to an accuracy of 88.2% (Fang et al., 2023). This demonstrates AI’s potent capability to mine novel, synergistic biomarker combinations from “stale” data—a strategy fully applicable to gastric cancer biomarker development.

##### 3.3.3.2 Explainable AI for integrated decision making

In clinical practice, physicians need not only results, but also an understanding of the rationale behind AI’s judgments. Explainable AI (XAI) was designed for this purpose. For example, in the liver cancer biomarker study mentioned, researchers used tools such as SHAP to clearly visualize the contribution of novel biomarkers such as SSBP3 and COX7A2L to the predictive model, making the “black box” model transparent and trustworthy (Willms et al., 2025). In the field of gastric cancer, when building multimodal AI integration systems that combine endoscopic images, clinical parameters, lifestyle data, and multiomics biomarkers (e.g., methylation and microbiome), XAI technology can provide clinicians with a visual, evidence-based decision support report. It clarifies which imaging features and molecular signals collectively indicate high risk, thereby facilitating its adoption and application in clinical settings (Willms et al., 2025).

#### 3.3.4 Pathways to technological inclusivity and ethical considerations

##### 3.3.4.1 Feasible pathways for promoting technological inclusivity

*Lightweight and mobile deployment*: Developing lightweight models and mobile assisted applications tailored to the hardware constraints of primary care settings can effectively lower the deployment threshold.

*Building a collaborative ecosystem*: Policy guidance is needed to promote the construction of regional medical AI cloud platforms that facilitate the flow and sharing of technology, data, and talent, all within a framework that ensures data privacy security and algorithmic ethics.

The dual support system of “artificial intelligence + biomarkers” steers gastric cancer prevention and control in a new era. AI acts not merely as a tool to enhance diagnostic sensitivity but also as a transformative force. Through its capabilities of standardization, replicability, and prospective early warning, it fundamentally addresses health care disparities. The deep integration of biomarkers with AI, particularly the transparency brought about by XAI and its ability to synthesize multidimensional data, lays a solid foundation for truly individualized and precise prevention. As this system continues to improve and, we anticipate a paradigm shift in gastric cancer control from “universal screening” to “precision risk adapted management,” ultimately achieving the public health goals of increasing early diagnosis rates and reducing mortality.

Ethical considerations of risk reclassification deserve explicit attention. In a risk-adapted pathway, some individuals may be reclassified from low to higher risk even after successful eradication of *H. pylori*, particularly when premalignant mucosal changes (e.g., atrophy or intestinal metaplasia), family history, or adverse lifestyle factors are identified. Clinicians should communicate that eradication reduces—but does not eliminate—gastric cancer risk, and use absolute-risk framing to avoid unnecessary alarm. Counseling should be grounded in shared decision-making, documenting the rationale for intensified surveillance, discussing potential benefits (earlier detection) and burdens (anxiety, procedural risks, costs), and offering psychosocial support and clear follow-up plans. At a system level, safeguards against inequitable access and unintended stigma should accompany reclassification, including transparent criteria, auditability, and privacy protection for risk data.

### 3.4 Resource allocation: differentiated deployment based on regional risk stratification

To achieve optimal resource allocation, prevention and control strategies must consider both regional gastric cancer incidence rates and economic development levels to establish differentiated resource allocation schemes. Primary care institutions should be equipped with basic serological testing devices to ensure regular follow-up for low-to-moderate-risk populations, whereas regional medical centers should be equipped with advanced endoscopic equipment to meet the precise examination needs of high-risk groups. This establishes a closed-loop management system of “primary screening → advanced diagnosis → primary follow-up.”

#### 3.4.1 We recommend categorizing implementation regions into the following four types and formulating corresponding prevention pathways

In regions with a high gastric cancer incidence and underdeveloped economies, priority should be given to ensuring the accessibility of basic screening services. Efforts should focus on popularizing non-invasive serological tests (e.g., pepsinogen ratio and Gastrin-17) in primary care institutions to establish a population-based initial screening network. Simultaneously, relying on regional medical centers for targeted support, endoscopic services should be provided for those who test positive in the initial screening, forming a tiered prevention and control system characterized by “broad coverage at the primary level and quality enhancement at the regional level.”

In regions with a high gastric cancer incidence and developed economies, it is recommended to build a comprehensive precision prevention network. On one hand, integrate risk stratification assessment for patients after *H. pylori* eradication into routine health management systems. On the other hand, high-definition endoscopy and AI-assisted diagnostic technology should be promoted in regional medical centers to achieve standardized monitoring of high-risk groups and precise identification of early lesions.

Resource allocation should be targeted to regions with a low incidence of gastric cancer. In economically developed low-incidence areas, leverage the existing healthcare system to focus on precise screening for individuals with clearly identified risk factors (e.g., heavy smoking, family history of gastric cancer, and confirmed precancerous lesions), avoiding unnecessary universal screening. In economically underdeveloped low-incidence areas, it primarily enhances public awareness through health education, and strengthens the identification and referral of relevant symptoms during clinical diagnosis and treatment.

Through this regional risk-stratification-based resource allocation model, the maximized utilization of limited medical resources can be ensured, enabling the precise implementation of prevention and control measures. Ultimately, this builds a gastric cancer prevention and control system tailored to the needs of each region.

## 4 Conclusion

The clinical value of *H. pylori* eradication therapy is undeniable; however, effective management of residual gastric cancer risk post-eradication has become a critical next step in advancing global prevention strategies. The comprehensive framework proposed in this study—integrating multidimensional risk assessment, stratified monitoring, and optimized resource allocation—provides a viable pathway for precision management in the post-eradication era. A cornerstone of this framework is the strategic incorporation of AI, which serves a dual role: as a precision tool for enhancing risk stratification and early detection through the analysis of endoscopic, clinical, and biomarker data and as an equity-enabling platform that can be deployed in resource-limited settings to standardize diagnosis and bridge healthcare disparities.

Future research should prioritize the validation of this AI-supported framework’s long-term cost-effectiveness, the development of more robust and explainable risk prediction models, and the discovery of novel biomarkers for early detection. It is only through such a systematic, technology-enhanced, and adaptively implemented risk-management strategy that we can fully address the complex challenge of residual gastric cancer risk and achieve a substantial reduction in its global burden.

As a concrete next step, we propose a prospective, multicentre pilot study enrolling approximately 1,000 post-eradication patients with baseline endoscopic and histologic staging, with a planned 3-year follow-up. Participants would be managed either with the proposed stratified protocol (risk-tiered surveillance intervals and adjunct biomarker/AI-assisted assessment) or with standard care as currently delivered at participating centres (e.g., uniform or guideline-minimum follow-up). The pilot should be powered to detect a 30% relative reduction in advanced-stage gastric cancer diagnoses (e.g., stages II–IV) in the stratified arm, with secondary endpoints including stage distribution, detection of early neoplasia, surveillance adherence, procedure-related harms, and cost-effectiveness. A pragmatic cluster-randomized or stepped-wedge implementation design would additionally allow evaluation of feasibility across heterogeneous healthcare settings.

## Data Availability

The original contributions presented in the study are included in the article/Supplementary material, further inquiries can be directed to the corresponding author.

## Supplementary material

Supplementary File S1 Risk calculator (Excel) for stratifying residual gastric cancer risk after *H. pylori* eradication.

Supplementary File S2 Web-based risk calculator (HTML) implementing the same scoring and risk-tier output as Supplementary File S1.

## References

[ref1] Borka Balas R. Melit L. E. Marginean C. O. (2022). Worldwide prevalence and risk factors of *Helicobacter pylori* infection in children. Children 9:1359. doi: 10.3390/children9091359, 36138669 PMC9498111

[ref2] Cheung K. S. Chan E. W. Wong A. Y. S. Chen L. Wong I. C. K. Leung W. K. (2018). Long-term proton pump inhibitors and risk of gastric cancer development after treatment for *Helicobacter pylori*: a population-based study. Gut 67, 28–35. doi: 10.1136/gutjnl-2017-314605, 29089382

[ref3] den Hoed C. M. Holster I. L. Capelle L. G. de Vries A. C. den Hartog B. Ter Borg F. . (2013). Follow-up of premalignant lesions in patients at risk for progression to gastric cancer. Endoscopy 45, 249–256. doi: 10.1055/s-0032-1326379 23533073

[ref4] Dinis-Ribeiro M. Areia M. de Vries A. C. Marcos-Pinto R. Monteiro-Soares M. O’Connor A. . (2012). Management of precancerous conditions and lesions in the stomach (MAPS): guideline from the ESGE/EHSG/ESP/SPED. Endoscopy 44, 74–94. doi: 10.1055/s-0031-1291491 22198778 PMC3367502

[ref5] Fang S. Liu Z. Qiu Q. Tang Z. Yang Y. Kuang Z. . (2023). Diagnosing and grading gastric atrophy and intestinal metaplasia using semi-supervised deep learning on pathological images: development and validation study. Gastric Cancer 27, 343–354. doi: 10.1007/s10120-023-01451-9, 38095766 PMC10896941

[ref6] Fontes F. Kapteijn N. E. A. Hassan C. Deane C. Cristiano M. Fernandes-Mendes H. . (2025). Adherence to clinical practice guidelines for management of epithelial precancerous conditions and lesions in the stomach in Europe. Endoscopy 57, 1338–1347. doi: 10.1055/a-2695-1376, 40902622 PMC12668271

[ref7] Gutierrez-Chakraborty E. Chakraborty D. Das D. Bai Y. (2024). Discovering novel prognostic biomarkers of hepatocellular carcinoma using eXplainable artificial intelligence. Expert Syst. Appl. 252:124239. doi: 10.1016/j.eswa.2024.124239, 39829683 PMC11737334

[ref8] Hirasawa T. Aoyama K. Tanimoto T. Ishihara S. Shichijo S. Ozawa T. . (2018). Application of artificial intelligence using a convolutional neural network for detecting gastric cancer in endoscopic images. Gastric Cancer 21, 653–660. doi: 10.1007/s10120-018-0793-2, 29335825

[ref9] Hu Y. Wan J.-H. Li X.-Y. Zhu Y. Graham D. Y. Lu N.-H. (2017). Systematic review with meta-analysis: the global recurrence rate of *Helicobacter pylori*. Aliment. Pharmacol. Ther. 46, 773–779. doi: 10.1111/apt.14319, 28892184

[ref10] Hu C. Xia Y. Zheng Z. Cao M. Zheng G. Chen S. . (2025). AI-based large-scale screening of gastric cancer from noncontrast CT imaging. Nat. Med. 31, 3011–3019. doi: 10.1038/s41591-025-03785-6, 40555751 PMC12443630

[ref11] Huang J. Q. Zheng G. F. Sumanac K. Irvine E. J. Hunt R. H. (2003). Meta-analysis of the relationship between cagA seropositivity and gastric cancer. Gastroenterology 125, 1636–1644. doi: 10.1053/j.gastro.2003.08.033, 14724815

[ref12] Li D. Jiang S. F. Lei N. Y. Shah S. C. Corley D. A. (2023). Effect of *Helicobacter pylori* eradication therapy on the incidence of noncardia gastric adenocarcinoma in a large diverse population in the United States. Gastroenterology 165, 391–401.e2. doi: 10.1053/j.gastro.2023.04.026, 37142201

[ref13] Li T. H. Qin Y. Sham P. C. Lau K. S. Chu K. M. Leung W. K. (2017). Alterations in gastric microbiota after *H. pylori* eradication and in different histological stages of gastric carcinogenesis. Sci. Rep. 7:44935. doi: 10.1038/srep44935, 28322295 PMC5359573

[ref14] Ligato I. Dottori L. Sbarigia C. Dilaghi E. Annibale B. Lahner E. . (2024). Systematic review and meta-analysis: risk of gastric cancer in patients with first-degree relatives with gastric cancer. Aliment. Pharmacol. Ther. 59, 606–615. doi: 10.1111/apt.17872, 38197125

[ref15] Luo H. Xu G. Li C. He L. Luo L. Wang Z. . (2019). Real-time artificial intelligence for detection of upper gastrointestinal cancer by endoscopy: a multicentre, case-control, diagnostic study. Lancet Oncol. 20, 1645–1654. doi: 10.1016/S1470-2045(19)30637-0, 31591062

[ref16] Machlowska J. Baj J. Sitarz M. Maciejewski R. Sitarz R. (2020). Gastric cancer: epidemiology, risk factors, classification, genomic characteristics and treatment strategies. Int. J. Mol. Sci. 21:4012. doi: 10.3390/ijms21114012, 32512697 PMC7312039

[ref17] Mera R. M. Bravo L. E. Camargo M. C. Bravo J. C. Delgado A. G. Romero-Gallo J. . (2018). Dynamics of *Helicobacter pylori* infection as a determinant of progression of gastric precancerous lesions: 16-year follow-up of an eradication trial. Gut 67, 1239–1246. doi: 10.1136/gutjnl-2016-311685 28647684 PMC5742304

[ref18] Miki K. (2006). Gastric cancer screening using the serum pepsinogen test method. Gastric Cancer 9, 245–253. doi: 10.1007/s10120-006-0397-0, 17235625

[ref19] Miki K. (2011). Gastric cancer screening by combined assay for serum anti-*Helicobacter pylori* IgG antibody and serum pepsinogen levels—“ABC method”. Proc. Jpn. Acad. B 87, 405–414. doi: 10.2183/pjab.87.405, 21785258 PMC3171284

[ref20] Monteiro E. J. M. Costa C. Oliveira J. L. (2016). A cloud architecture for teleradiology-as-a-service. Methods Inf. Med. 55, 203–214. doi: 10.3414/ME14-01-0052, 26940635

[ref21] Pimentel-Nunes P. Libanio D. Marcos-Pinto R. Areia M. Leja M. Esposito G. . (2019). Management of epithelial precancerous conditions and lesions in the stomach (MAPS II): ESGE/EHMSG/ESP/SPED guideline update 2019. Endoscopy 51, 365–388. doi: 10.1055/a-0859-1883, 30841008

[ref22] Robles C. Rudzite D. Polaka I. Sjomina O. Tzivian L. Kikuste I. . (2022). Assessment of serum pepsinogens with and without co-testing with gastrin-17 in gastric cancer risk assessment—results from the GISTAR pilot study. Diagnostics 12:1746. doi: 10.3390/diagnostics12071746, 35885649 PMC9325279

[ref23] Salvatori S. Marafini I. Laudisi F. Monteleone G. Stolfi C. (2023). *Helicobacter pylori* and gastric cancer: pathogenetic mechanisms. Int. J. Mol. Sci. 24:2895. doi: 10.3390/ijms24032895, 36769214 PMC9917787

[ref24] Serelli-Lee V. Ling K. L. Ho C. Yeong L. H. Lim G. K. Ho B. . (2012). Persistent *Helicobacter pylori* specific Th17 responses in patients with past *H. pylori* infection are associated with elevated gastric mucosal IL-1beta. PLoS One 7:e39199. doi: 10.1371/journal.pone.0039199 22761739 PMC3382622

[ref25] Shi Y. Wei N. Wang K. Wu J. Tao T. Li N. . (2023). Deep learning-assisted diagnosis of chronic atrophic gastritis in endoscopy. Front. Oncol. 13:1122247. doi: 10.3389/fonc.2023.1122247, 36950553 PMC10025314

[ref26] Su C.-H. Islam M. M. Jia G. Wu C.-C. (2022). Statins and the risk of gastric cancer: a systematic review and meta-analysis. J. Clin. Med. 11:7180. doi: 10.3390/jcm11237180, 36498753 PMC9739712

[ref27] Sugano K. Tack J. Kuipers E. J. Graham D. Y. El-Omar E. M. Miura S. . (2015). Kyoto global consensus report on *Helicobacter pylori* gastritis. Gut 64, 1353–1367. doi: 10.1136/gutjnl-2015-309252, 26187502 PMC4552923

[ref28] Thrift A. P. Wenker T. N. El-Serag H. B. (2023). Global burden of gastric cancer: epidemiological trends, risk factors, screening and prevention. Nat. Rev. Clin. Oncol. 20, 338–349. doi: 10.1038/s41571-023-00747-0, 36959359

[ref29] Verbraak F. D. Abramoff M. D. Bausch G. C. F. Klaver C. Nijpels G. Schlingemann R. O. . (2019). Diagnostic accuracy of a device for the automated detection of diabetic retinopathy in a primary care setting. Diabetes Care 42, 651–656. doi: 10.2337/dc18-0148, 30765436

[ref30] Wang Y. Wang X. Cao X.-Y. Zhu H.-L. Miao L. (2023). Comparative effectiveness of different probiotics supplements for triple *helicobacter pylori* eradication: a network meta-analysis. Front. Cell. Infect. Microbiol. 13:1120789. doi: 10.3389/fcimb.2023.1120789, 37256113 PMC10226649

[ref31] Wang Y. Zhu Z. Liu Z. Zhao Z. Xue X. Li X. . (2020). Diagnostic value of serum pepsinogen I, pepsinogen II, and gastrin-17 levels for population-based screening for early-stage gastric cancer. J. Int. Med. Res. 48:300060520914826. doi: 10.1177/0300060520914826, 32228342 PMC7132564

[ref32] Wiklund A. K. Santoni G. Yan J. Radkiewicz C. Xie S. Birgisson H. . (2025). Risk of gastric adenocarcinoma after eradication of *Helicobacter pylori*. Gastroenterology 169, 244–250.e1. doi: 10.1053/j.gastro.2025.01.239, 39924057

[ref33] Willms A. G. Krawczyk M. Julich-Haertel H. Gries S. K. Banales J. M. Mocan T. . (2025). What we missed then, AI sees now: revisiting legacy large extracellular vesicle data to reveal synergistic biomarkers for liver cancer screening. JHEP Rep. 7:101540. doi: 10.1016/j.jhepr.2025.101540, 41127881 PMC12538156

[ref34] Wu L. Shang R. Sharma P. Zhou W. Liu J. Yao L. . (2021). Effect of a deep learning-based system on the miss rate of gastric neoplasms during upper gastrointestinal endoscopy: a single-centre, tandem, randomised controlled trial. Lancet Gastroenterol. Hepatol. 6, 700–708. doi: 10.1016/S2468-1253(21)00216-8, 34297944

[ref35] Yamada H. Abe S. Charvat H. Ando T. Maeda M. Murakami K. . (2025). Precision risk stratification of primary gastric cancer after eradication of *H. pylori* by a DNA methylation marker: a multicentre prospective study. Gut 74, 1410–1418. doi: 10.1136/gutjnl-2025-335039, 40240063 PMC12418579

[ref36] Yan H. Li Z. Zhao J. Su L. Jin Z. Zhao W. . (2025). AI-assisted detection of early gastric cancer via visualization of mucosal acidity compromise during endoscopy. Adv. Sci. 12:e202504932. doi: 10.1002/advs.202504932, 41114471 PMC12697849

[ref37] Yang Z. Zhou Y. Han Z. He K. Zhang Y. Wu D. . (2024). The effects of probiotics supplementation on *Helicobacter pylori* standard treatment: an umbrella review of systematic reviews with meta-analyses. Sci. Rep. 14:10069. doi: 10.1038/s41598-024-59399-4, 38697990 PMC11066092

