# Supplementary material for: Stratified management of residual gastric cancer risk after Helicobacter pylori eradication
Source: Front Microbiol. 2026 Feb 13;17:1779490. doi: 10.3389/fmicb.2026.1779490 (PMC12946149; doi:10.3389/fmicb.2026.1779490)
Supplement: Supplementary File S1 — Risk calculator (Excel) for stratifying residual gastric cancer risk after H. pylori eradication. [file Data_Sheet_1.ZIP › Supplementary_File_S2_Risk_Calculator_Web.html]

Mock-up Post-H. pylori Residual GC Risk Calculator


# Mock-up Multidimensional Risk Calculator (Supplementary File S2)

Illustrative tool for applying the manuscript’s post-eradication risk framework (not a validated prediction model).
Mucosal status uses the **highest OLGA/OLGIM stage** (0–IV), consistent with guideline concepts (MAPS-II / Kyoto).

Total score

0

Other high-risk factors (count)

0

Risk tier (per framework)

Low-to-moderate risk

**Illustrative management suggestion:** Lifestyle optimization; routine care per local guidance

## Point breakdown

| Variable | Selection | Points | Notes |
| --- | --- | --- | --- |

Note: This mock-up operationalizes the framework described in the manuscript. It is intended for demonstration and should be
adapted/validated before clinical use.
